# Supplementary figures and images for: Estimating the disease burden of Korean type 2 diabetes mellitus patients considering its complications
Source: PLoS One. 2021 Feb 8;16(2):e0246635. doi: 10.1371/journal.pone.0246635 (PMC7870056; doi:10.1371/journal.pone.0246635)

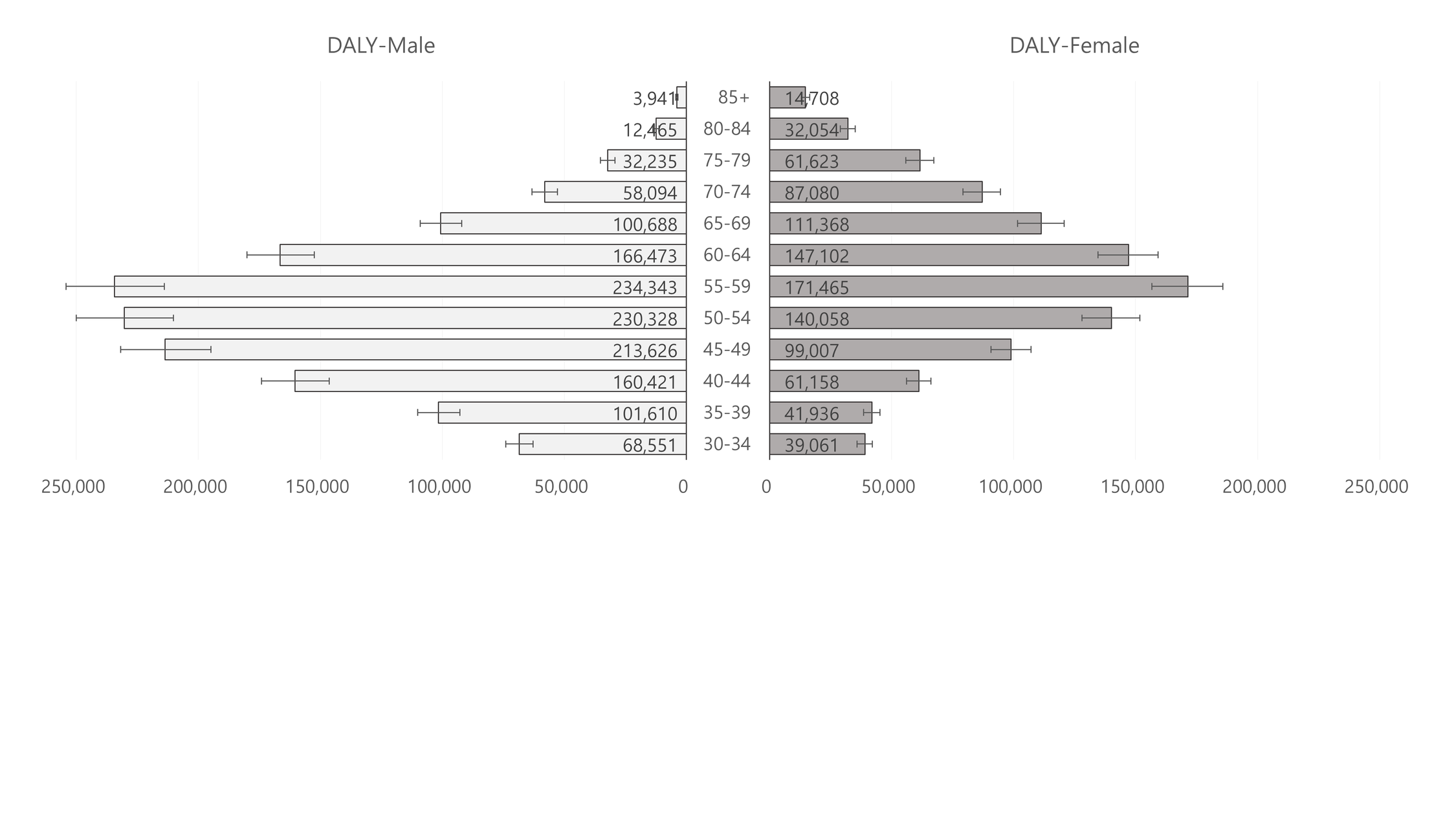

Supplement: S1 Fig — DALYs, disability-adjusted life years. (TIF) [file pone.0246635.s001.tif]

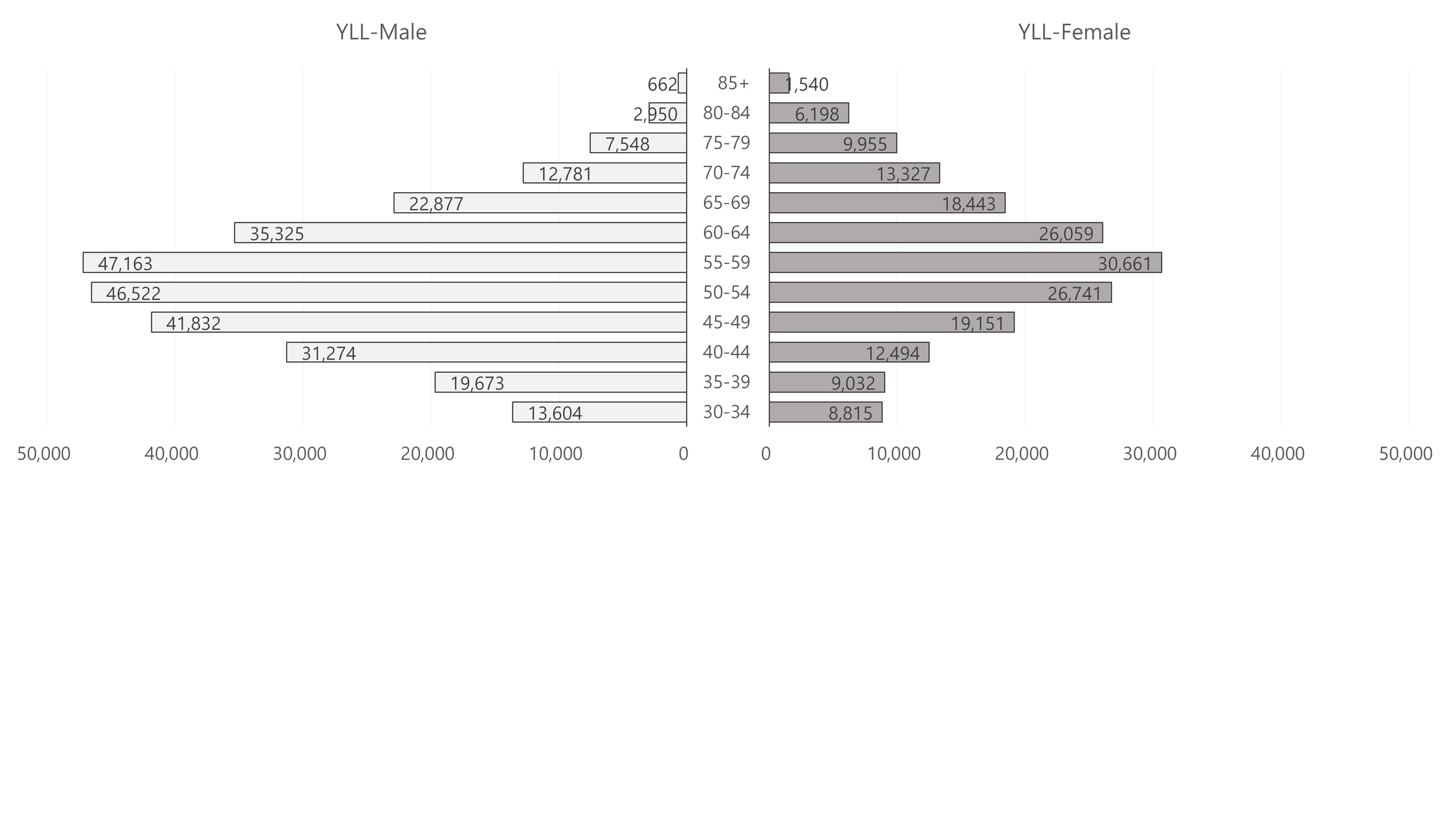

Supplement: S2 Fig — YLLs, years of life lost. (TIF) [file pone.0246635.s002.tif]

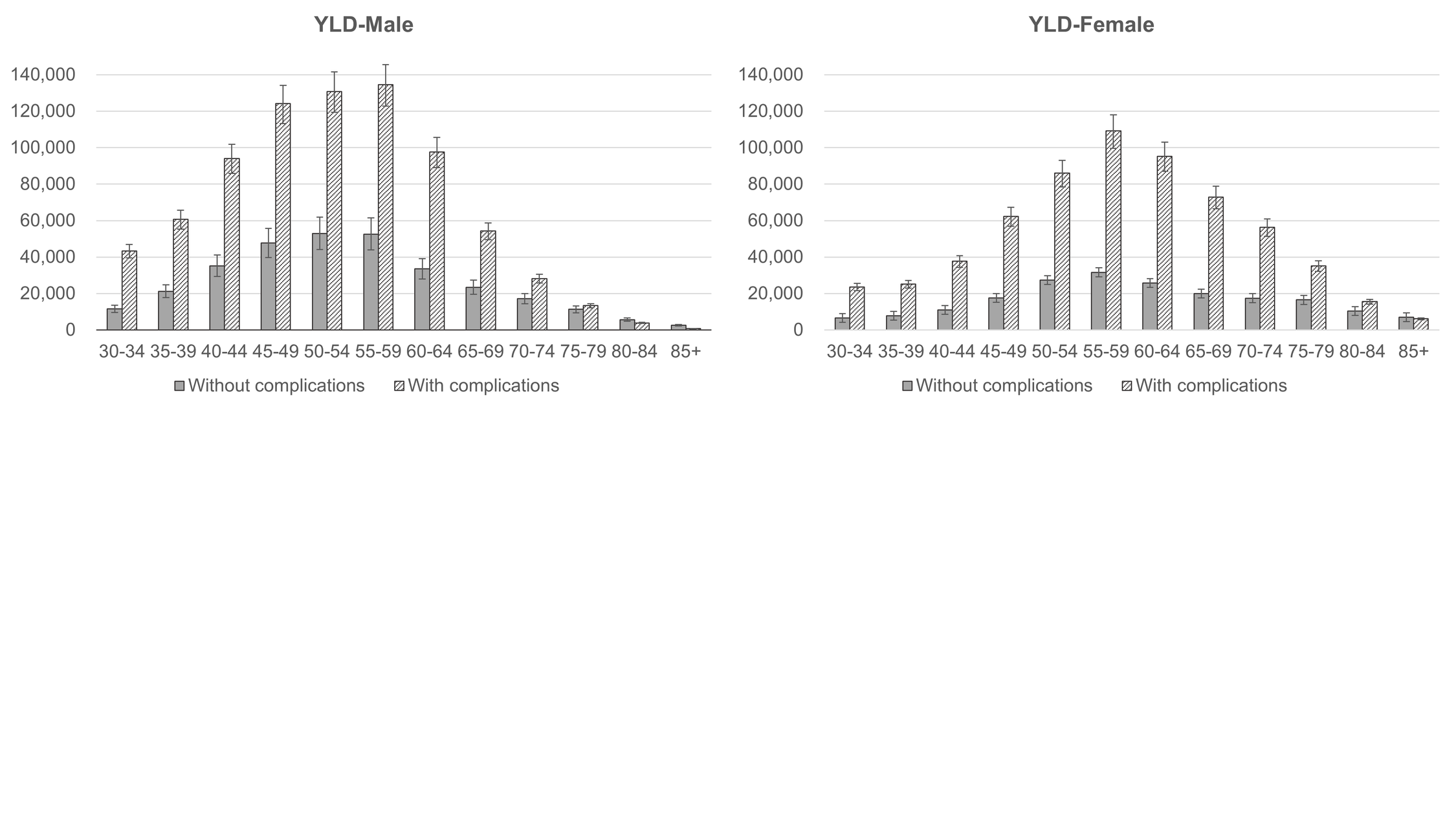

Supplement: S3 Fig — YLDs, years lived with disability. (TIF) [file pone.0246635.s003.tif]
