# Supplementary material for: Estimating the disease burden of Korean type 2 diabetes mellitus patients considering its complications
Source: PLoS One. 2021 Feb 8;16(2):e0246635. doi: 10.1371/journal.pone.0246635 (PMC7870056; doi:10.1371/journal.pone.0246635)
Supplement: S1 Table — (DOCX) [file pone.0246635.s004.docx]

S1 Table. Ingredient code of anti-diabetic drugs.

| **Anti-diabetic drugs** |
| --- |
| 165702ATB, 165801ATB, 165602ATB, 191502ATB, 165402ATB, 165701ATB, 191501ATB, 191503ATB, A0096801, 86101ATB, 132001ATB, 430201ATB, 430202ATB, 430203ATB, A0096601, 191504ATB, 249002ATB, 165704ATB, 249001ATB, 165602ACS, 165601ACS, 165401ATB, 165703ATB, 474200ATB, 474300ATB, 165901ATB, 165603ATR, A0097001, 421100ATB, 417402ATB, 417401ATB, B0111701, 170101BIJ, B0027001, B0027101, B0163201, B0163301, 170103BIJ, 118302BIJ, 215602BIJ, 118301BIJ, 215601BIJ, A0237601, A0237501, 175201BIJ, 175202BIJ, 215603BIJ, 165501ATB, 167701AGN, 191502AGR, 327800BIJ, 100602ATB, 100601ATB, 406201ATB, 406202ATB, 170402BIJ, 170102BIJ, 175301BIJ, 175302BIJ, 431901ATB, 431902ATB, 175303BIJ, 175304BIJ, 348001ATB, 348002ATB, 348003ATB, 452700ATB, 452900ATB, 461200ATB, 469100ATB, 471800ATB, 443400ATB, 443500ATB, 471900ATB, 191502ATR, 461802BIJ, 461801BIJ, 484902BIJ, 484901BIJ, 170602BIJ, 170502BIJ, 170403BIJ, 170302BIJ, 170201BIJ, 215701BIJ, 170401BIJ, 379503ATB, 379502ATB, 379501ATB, 441302BIJ, 441301BIJ, 441303BIJ, 170601BIJ, 170501BIJ, 170301BIJ, 488701BIJ, 488800ATB, 488900ATB, 489000ATB, 249001ATD, 249002ATD, 523600ATB, 523700ATB, 191504ATR, 474300ATR, 498600ATB, 525901ATB, 518800ATB, 527301ATB, 527302ATB, 512102BIJ, 512101BIJ, 626601BIJ, 626602BIJ, 616401ATB, 520700ATB, 520600ATB, 520500ATB, 500801ATB, 507000ATB, 507100ATB, 519600ATB, 441305BIJ, 441304BIJ, 501101ATB, 501102ATB, 501103ATB, 513700ATB, 502300ATB, 502900ATB, 513700ATR, 502300ATR, 524700ATR, 191505ATR, 613301ATB, 613302ATB, 518500ATR, 518600ATR, 497200ATB, 619101ATB, 523800ATR, 498100ATB, 507401BIJ, 165604ATR, 624202ATB, 624203ATB, 624201ATB, 525600ATB, 525500ATB, 630300ATB, 630400ATB, 630500ATB, 630600ATB, 645301ATB, 650100ATR, 650000ATR, 649900ATR, 631900ATB, 644900ATB, 639601ATB, 648400ATB, 648500ATB, 648600ATB, 644502BIJ, 644501BIJ, 512131BIJ, 512130BIJ, 639800ATR, 641400ATR, 461831BIJ, 484931BIJ, 461830BIJ, 484930BIJ, 626631BIJ, 626630BIJ, 461804BIJ, 461832BIJ, 627301ATB, 641800ATR, 641900ATR, 642000ATR, 628201ATB, 628202ATB, 441331BIJ, 441330BIJ, 441332BIJ, 488730BIJ, 170431BIJ, 170131BIJ, 441333BIJ, 441334BIJ, 626801BIJ, 626830BIJ, 626802BIJ, 626831BIJ, 632100ATB, 637200ATB, 632000ATR, 645000ATR, 175332BIJ, 175331BIJ, 175330BIJ, 170130BIJ, 170430BIJ, 175333BIJ, 639701BIJ, 639702BIJ, 636101ATB, 635700ATB, 635600ATB, 653900ATR, 653800ATR, 654000ATR, 655700ATR, 654100ATR, 502200ATB, 649000ATB, 649100ATB, 649200ATB, 649300ATB, 649400ATB, 649500ATB, 626700BIJ, 664600ATB, 664700ATB, 664800ATB |
