# Supplementary material for: Estimating the disease burden of Korean type 2 diabetes mellitus patients considering its complications
Source: PLoS One. 2021 Feb 8;16(2):e0246635. doi: 10.1371/journal.pone.0246635 (PMC7870056; doi:10.1371/journal.pone.0246635)
Supplement: S2 Table — ICD, International Classification of Disease. (DOCX) [file pone.0246635.s005.docx]

S2 Table. ICD-10 codes of diabetic complications.

| **ICD-10** | **Complication** | **Over 60** |
| --- | --- | --- |
| G632 | Diabetic polyneuropathy | Y |
| G990 | Autonomic neuropathy in endocrine and metabolic diseases | Y |
| H280 | Diabetic cataract | Y |
| H360 | Diabetic retinopathy | Y |
| N083 | Glomerular disorders in diabetes mellitus | Y |
| E112 | Type 2 diabetes mellitus, with renal complications | N |
| E113 | Type 2 diabetes mellitus, with ophthalmic complications | N |
| E114 | Diabetes mellitus, with neurological complications | N |
| E117 | Type 2 diabetes mellitus, with multiple complications | N |
| E122 | Malnutrition-related diabetes mellitus, with renal complications | N |
| E123 | Malnutrition-related diabetes mellitus, with ophthalmic complications | N |
| E124 | Malnutrition-related diabetes mellitus, with neurological complications | N |
| E127 | Malnutrition-related diabetes mellitus, with multiple complications | N |
| E132 | Other specified diabetes mellitus, with renal complications | N |
| E133 | Other specified diabetes mellitus, with ophthalmic complications | N |
| E134 | Other specified diabetes mellitus, with neurological complications | N |
| E137 | Other specified diabetes mellitus, with multiple complications | N |
| E142 | Unspecified diabetes mellitus, with renal complications | N |
| E143 | Unspecified diabetes mellitus, with ophthalmic complications | N |
| E144 | Unspecified diabetes mellitus, with neurological complications | N |
| E147 | Unspecified diabetes mellitus, with multiple complications | N |
| G45 | Transient cerebral ischaemic attacks and related syndromes | Y |
| I12 | Hypertensive renal disease | Y |
| I13 | Hypertensive heart and renal disease | Y |
| I20 | Angina pectoris | Y |
| I21 | Acute myocardial infarction | Y |
| I22 | Subsequent myocardial infarction | Y |
| I23 | Subsequent myocardial infarction | Y |
| I24 | Other acute ischaemic heart diseases | Y |
| I25 | Chronic ischaemic heart disease | Y |
| I61 | Intracerebral haemorrhage | Y |
| I62 | Other nontraumatic intracranial haemorrhage | Y |
| I63 | Cerebral infarction | Y |
| I64 | Stroke, not specified as haemorrhage or infarction | Y |
| I690 | Sequelae of subarachnoid haemorrhage | Y |
| I692 | Sequelae of other nontraumatic intracranial haemorrhage | Y |
| I693 | Sequelae of cerebral infarction | Y |
| I694 | Sequelae of stroke, not specified as haemorrhage or infarction | Y |
| N18 | Chronic kidney disease | Y |
| N183 | Chronic kidney disease, stage 3 | Y |
| N184 | Chronic kidney disease, stage 4 | Y |
| N185 | Chronic kidney disease, stage 5 | Y |
| N19 | Chronic kidney disease, unspecified | Y |

ICD, International Classification of Disease.
