# Supplementary material for: Estimating the disease burden of Korean type 2 diabetes mellitus patients considering its complications
Source: PLoS One. 2021 Feb 8;16(2):e0246635. doi: 10.1371/journal.pone.0246635 (PMC7870056; doi:10.1371/journal.pone.0246635)
Supplement: Attachment — Submitted filename: Response to Reviewers_FI.docx [file pone.0246635.s006.docx]

Brecht Devleesschauwer, MD, PhD

Academic Editor

PLOS ONE

Submitted: Sep 7, 2020

Dear Dr. Devleesschauwer:

Thank you for inviting us to submit a revised draft of our manuscript entitled, " Estimating the disease burden of Korean type 2 diabetes mellitus patients considering its complications " to the PLOS ONE. We also greatly appreciate the time and effort you and each of the reviewers have dedicated to providing insightful comments on ways to strengthen our paper. Thus, it is with great pleasure that we resubmit our article for further consideration. We have incorporated changes that reflect the detailed suggestions you have graciously provided. We also hope that our edits and the responses we provide below satisfactorily address all the issues and concerns you and the reviewers have noted.

To facilitate your review of our revisions, the following is a point-by-point response to the questions and comments delivered in your letter dated Oct 15, 2020.

| **Requirements** | **Authors’ response** |
| --- | --- |
| Journal Requirements | We uploaded copies of four figures including Fig 4.  The changes highlighted in two ways: grammar/spelling errors (shadow) and others (blue) |
| Additional Editor Comments | Our manuscript has been proofread by Editage ([www.editage.co.kr](http://www.editage.co.kr)). |

Reviewer 1

| **Reviewer 1 comment** | **Authors’ response** |
| --- | --- |
| While national burden of disease studies are of value, since they are based on known data from national data sources, and may be more comfortable to national policy makers, they are not comparable to the results produced in the global GBD study. Thus publishing national studies in international journals is hardly meaningful. It could have been interesting to compare findings from this study with findings using the data on South Korea from the international burden of disease database, but this is not done. Furthermore, the specific aims of this study, even for national purposes, is not clear. There is no comparison to other countries, there is no comparison to other diseases, there is no data on risk factors (that are available in the GBD study), So the interest of these findings for policy making is not clear. | Thank you for providing these insights. We have redrafted the introduction (p.4, lines 66-68) and discussion section (p.17, lines 270-276) to establish a clear focus. We hope these changes will convince you. As mentioned in our manuscript, DALY also can be used in cost effective analyses (CEA) and a Markov model is also widely used in economic evaluations. It means that DALY estimation can conducted in the process of CEA, simultaneously. |
| While the data sources used are appropriate, and the application of sequalae of Diabetes type 2 is appropriate, and in line with the GBD study, I can not judge the Markov methodology to arrive at the overall burden of disease. While probably statistically well done, the authors do not give a convincing argument for the use of this methodology. I can only note that while the prevalence of diabetes type 2 seems quite in line with international studies, the number of DALYs/100000 is much higher than those obtained in the GBD study. See e.g. Khan, Hashim et al Int J Epidemiology and Global Health Volume 10, Issue 1, March 2020, Pages 107 – 11. This and other publications in the global network assessing burden of disease due to diabetes type 2 should be referenced. | We have reflected this comment by adding the comparisons with previous studies you mentioned (p.18, lines 307-310) . We agree that your point; however, we believe that previous DALY outcomes may be underestimated considering the research by Cho et al. (2019). They pointed out the shorter disease duration calculated by DisMod II than the Markov model. |

Reviewer 2

| **Reviewer 2 comment** | **Authors’ response** |
| --- | --- |
| Methods section:  In general, there are few methodological choices that are lacking an explanation.  • At pag 4 when you say: “Initial states were assumed to stay in their states for a defined single cycle, then move to other Markov states, including themselves.” What do you mean with “including themselves”? That they can stay in the same health state? Please rephrase | We have clarified our methodological choices throughout the section of materials and methods.  We have revised the text (p.4, lines 81-86) reflecting your comments by adding the transition example. |
| • Why use an age limit of 30 y ears for identification of T2DM cases | We have added the explanation about the reason why we limit the age in the text (p.6 lines 119-121). |
| • Could you please provide a better explanation of your approach for the incident and prevalent cases on pag 5 | We have revised the text (p.6 lines 121-126) to reflect your comment. |
| • Could you maybe describe what do you mean for washout period for the case definition? and why do you use it | We have added the explanation and the reference to this comment (p.6 lines 121-124) in connection with the operational definition of incident cases. |
| • Are the causes of deaths also retrieved from the NHIS? | Yes, we obtained the causes of deaths from the NHIS-NSC, which is sample cohort. While the NHIS database, including the whole Korean population, do not have the cause of death, the NHIS-NSC was established with the information of the causes of death. The reference [23] is the cohort profile of NHIS-NSC. |
| • Could you please describe better the process used for th e sensitivity analysis? Why did you choose specifically that subcategories? What was the process? I don’t understand the rationale in modifying the disease duration just for that subcategory. | We have added the explanation of the reverse transition (p.8, lines 169-178), which is subjected to the sensitivity analysis in the methods section. |
| Results section:  • Could you please remind the year of reference when you describe the results? Is 2016 for both incidence and prevalence? | We clarified what you pointed out. We had added the number of incident cases in 2016. And we more clarified the incidence rate we used for the transition rate in the Markov model was between 2009 and 2015 (p.9, lines 191-196). |
| • “While there was an increasing tendency in the incidence rates with age, the age specific diabetes incidence rate exceeded the aggregate incidence rate (12.5 per 1,000 people) from the 50–54 age group in men.” I would change the first part of the sentence. In the way it is written it seems like you are describing 2 opposite trends. | Thank you for your suggestion. I rephrased the sentence (p.9, lines 196-197). |
| • Pag 9 – “The occurrence probabilities of complications in the incident cases of diabetes were highe r than in the existing prevalent cases of diabetes in relatively young age groups in both genders.” This is interesting but I would think about another way of writing the concept. I don’t see the reversed tendency expressed in the sentence that is following. | Thank you for your suggestion. I rephrased the sentence (p.12, lines 213-217). |
| • I would not use the term “mountain shapes”. Please switch to bell shape or normally distributed. | Thank you for your suggestion. I rephrased the sentence (p.13, line 248). |

Reviewer 3

| The article will improve enormously if the methodology is clearer described. The way the article is written, it is understandable only for insiders and people with deep knowledge in the topic. In order to be understood from a broader audience, some of the terminology should be further explained. For instance, in simpler language the Markov model should be explained where the terminology should be clearer: Markov states, cycles, discount rates, etc. | Thank you for your suggestion. We have clarified that you mentioned thoughout the section of materials and methods. |
| --- | --- |
| Furthermore, many assumptions in the model (pages 6 and 7) stay unclear why they are taken. Better descriptions and arguments must be brought up. | We have revised the the methods section and added up more detailed explanations regarding our assumption (p.5, lines 92-95, p.6-8). |
| There is a mismatch between the numbering of the graphs. The supporting files have other numbers than the one in the text. | Thank you for your suggestion. We have renumbered of the figures. |
| In my opinion, the article also needs English proof. Some of the sentences are not really clear. | Thanks, Our manuscript has been proofread by Editage ([www.editage.co.kr](http://www.editage.co.kr)). |
| Further suggestions:  Page 3, Introduction, first sentence: “diabetes is leading cause, accounting …” –> is this globally meant or for Korea? Second sentence: (…) jumped from 18th to 11th between (…) -> not clear what this is. Is this place of ranking? | Thank your for the comment. After checking the refernece again, We revised the sentence (p.3, lines 42-43). |
| Third sentence and fourth sentence have a repetition: “Considering substantial prevalence of diabetes, the diabetes burden is expected to be greater in the future” and then “(…) the burden of diabetes is expected to increase, giving increasing prevalence.” | We would like to metion about the global trend in the third and want to narrow down to Korean context from the fourth sentence. We have revised the third sentence (p.3, line 47). |
| Fifth sentence: “(…) T2DM dominates the vast majority so it accounts for (…)” -> the vast majority of what? | We have revised the text (p.3, lines 51-53) to clarify the sentence. |
| Page 3, Paragraph 2: here it is mentioned DisMod II for the first time. Many readers would not know what it is, who has developed it, how it functions. A conventional tool is not really the right description; the access to it is very limited. Disease period -> is here duration meant? And why it should be important for diabetes, when this disease is irreversbale? | You have asked an interesting question. DisMod II was used not only in the GBD study but also in the KNBD study. We also mentioned about DisMod II in Discussion section (p.17, p.18). Cho et al. (2019) showed shorter morbid duration obatined from DisMod II, compared to a Markove model. |
| Page 4, paragraph 1: As mentioned above, several terminologies should be better explained.  Sentence: “Initial states were assumed (…), including themselves. -> please edit, it is not clear what is meant.  Sentence after: why are diabetes and diabetic states assumed to be irreversible? -> they are irreversible, there is no place for assumptions here. | We have revised the text (p.4, lines 81-86) reflecting your comments by adding the transition example.  We have also added explanations regarding the reason why we did not reversible states from diabetes (p.4-5, lines 89-92). |
| Sentence next: why is the assumption for five cycles? What is the justification? | We have added explanations regarding our assumption (p.5, lines 92-95). |
| Sentence next: “Among the 12 conceptual transitions (…)” -> which are these 12 transitions? | Thanks for comments. When we count one death state, it was 12 states. We revised this to 16 conceptual transtions by seperating death states to ‘due to diabetes’ and ‘due to other causes’ (p.5, lines 95). |
| Page 6, paragraph 1: Here should be explained what a washout period is and why a three-year washout period was assumed? What is the justification for this? | A three-year washout period was chosen based on the previous research. Roberts et al (2015) showed a three-year is appropriate to obtain true incident cases from claim data (p.6, lines 123-124). |
| Page 6, formula: Is time here meant to be duration? | Yes, it is. We have added up information regarding the formula (p.7, line 145). |
| Page 6, last sentence: should be explained what smoothing methods imply and why they are used. | We have rvised the text and given more explanation (p.7, lines 152-153). |
| Page 7, paragraph 1: Not clear why there was a replacement in case a mortality probability was less than the general mortality probability. And why in age group 30-34 zero mortalities were replaced with general death probabilities? | We have rvised the text and given more explanation (p.7, lines 156-158). |
| Page 7, paragraph 2: “(…) by the duration between transitions of interest” -> what are the transitions of interest here? | We have rvised the text and given more explanation (p.8, lines 162-163). |
| Page 7, last paragraph: “A sensitivity analysis was conducted for the reverse transitions (…)” -> what are here reverse transitions in case of diabetes? | We have rvised the text and given more explanation (p.8, lines 169-178). In our Markov model, diabetic complications occur in diabetic patients becasue we only consider diabetic incident cases as the initial states. In other words, diabetic complications cannot be occur before the incidence of diabetes. We used caim data (administrative data), so we should deal with this issue. |
| Page 8, paragraph 1, last sentence: University name not readable. | We have revised the text (p.9, line 188). |
| Page 12, Tables 2A and 2B: In the methods section it is written that sensitivity analysis is performed for ages 60 +. Why these tables include information on younger age groups? There are also no differences in the numbers there. | We have revised the methods section (p.8, lines 169-178). In the sensitivity analysis, we only deal with diabetic complications (other than E codes, occurred over 60 years of age) such as stroke. In terms of diabetic complications (ICD-10 E.** codes), these are obivous diabetic complications. So diabetes codes (E11-E14) would have omitted by the mistake or coding patterns. |
| Page 13, paragraph 1: “The disease burden (…) than that in the previous KNBD study -> please include a reference here, even of it is repeating. | We have revised the text (p.17, line 273). |
| Page 15, last sentence: “three years of experience” -> I don’t think “experience” here is the right word. | We have revised the word to ‘morbid duration’ (p.20, line 344). |
| Figures 2 and 3 in the text (in the files they have the names Figure 1 and 2): These figures look like age pyramids and intuitively one expects the youngest age groups to start from the bottom and go up. Consider to change it. | We have revised the figures as you suggested (Fig 2, Fig3, S1 Fig, S2 Fig). |
| Files Fig4.tif and S Fig3.tif: the figures have the same labels but present different results. | We have presented DALYs/YLLs per 100,000 population as the main outcomes (Fig2, Fig3). S Fig1 are S Fig2 are total numbers of Korean populations, additionally presented as supplementary figures. Therefore, these figures seem to be simiar to the Fig2 and Fig 3, but units are different. |

Again, thank you for giving us the opportunity to strengthen our manuscript with your valuable comments and queries. We have worked hard to incorporate your feedback and hope that these revisions persuade you to accept our submission.

Sincerely,

Min-Woo Jo, MD, PhD
Professor, Department of Preventive Medicine, University of Ulsan
Department of Preventive Medicine, University of Ulsan College of Medicine, 88, OLYMPIC-RO 43-GIL, SONGPA-GU, SEOUL 05505, KOREA
mdjominwoo@gmail.com
82-2-3010-4264
82-2-477-2898

Additional contact
Juyoung Kim, MSc
PhD candidate, Department of Preventive Medicine, University of Ulsan
Department of Preventive Medicine, University of Ulsan College of Medicine, 88, OLYMPIC-RO 43-GIL, SONGPA-GU, SEOUL 05505, KOREA
juyoungkim0716@gmail.com
82-2-3010-4809
82-2-477-2898
